# Ligature-induced peri-implant infection in crestal and subcrestal implants: a clinical and radiographic study in dogs

**DOI:** 10.7717/peerj.1139

**Published:** 2015-07-30

**Authors:** Baoxin Huang, Muzi Piao, Li Zhang, Xian’e Wang, Li Xu, Weidong Zhu, Huanxin Meng

**Affiliations:** 1 Department of Oral Implantology, Guanghua School of Stomatology, Hospital of Stomatology, Sun Yat-sen University, Guangzhou, China; 2 Guangdong Provincial Key Laboratory of Stomatology, Guangzhou, China; 3 Department of Periodontology, Peking University School and Hospital of Stomatology, Beijing, China; 4 The Second Dental Center, Peking University School and Hospital of Stomatology, Beijing, China

**Keywords:** Dental implants, Peri-implantitis, Bone resorption, Peri-implant defect, Dental implant–abutment design, Implant surface, Animals, Crestal remodeling, Subcrestal positioning

## Abstract

**Objective.** The aim of this study was to assess the influence of implant–abutment interface (IAI) placement depths on peri-implant tissues in the presence of ligature-induced peri-implant inflammation.

**Materials and Methods.** Two implants with screwed-in IAIs (SI) and two implants with tapped-in IAIs (TI) were inserted in one side of the mandible in six dogs eight weeks after tooth extraction. Four experimental groups were constituted: SI placed crestally, SI placed 1.5 mm subcrestally, TI placed crestally and TI placed 1.5 mm subcrestally. After 12 weeks, the healing abutments were connected. Four weeks later, cotton floss ligatures were placed around the abutments to promote plaque accumulation. Clinical and radiographic examinations were performed at 0, 6 and 12 weeks after ligature placement. The effects of the IAI placement depths on clinical and radiographic parameters were assessed.

**Results.** The alterations of peri-implant probing depths, clinical attachment levels, distances from the IAI to the first bone-implant contact (IAI-fBIC) and depths of infrabony defect were significant larger in the subcrestal groups compared with the crestal groups during the plaque accumulation period. The alterations of clinical attachment levels, IAI-fBIC, depth of the infrabony defect and horizontal bone loss were not significantly different between the SI and TI groups after ligature placement.

**Conclusion.** Tissue destruction in subcrestal implants may be more serious than that in crestal implants in the presence of inflamed peri-implant mucosa.

## Introduction

The replacement of a missing tooth by a dental implant has become a widely used treatment modality. In esthetic areas, subcrestal implant placement was recommended to minimize the risk of metal exposure and provide enough space in the vertical dimension to create an ideal emergence profile (Barros et al., 2010; Buser & Von Arx, 2000). In two-piece implants, however, increased soft tissue inflammation and crestal bone resorption were reported to be associated with subcrestal placement of the implant abutment interface (IAI) (Broggini et al., 2006; Hermann et al., 2000; Hermann et al., 1997). The results from experimental studies by Hermann et al. (2000) and Hermann et al. (1997) showed that crestal bone remodeling occurred ≈1.5 to 2 mm below the IAI. Broggini et al. (2006) found that the inflammatory reaction of peri-implant soft tissues progressively increased as the IAI depth increased. Moreover, another clinical study reported that moving the IAI coronally from the alveolar crest was correlated with fewer inflammatory markers such as interleukin-1 beta and tumor necrosis factor-alpha (Boynuegri et al., 2012). With this in mind, supracrestal IAI placement was recommended. Conversely, recent experimental and clinical studies using implants with tapered internal IAIs showed that subcrestal implant placement had a positive impact on crestal bone preservation around the cervix of the implant (Barros et al., 2010; Donovan et al., 2010; Huang et al., 2012). Meanwhile, the peri-implant epithelial dimension and biologic width were significantly greater for subcrestal implants compared with equicrestal or supracrestal implants (Cochran et al., 2013). Notably, the influence of the implant depth on peri-implant bone remodeling and soft tissue dimensions was evaluated under well-controlled oral hygiene conditions in previous studies (Barros et al., 2010; Cochran et al., 2013; Huang et al., 2012). Peri-implant inflammation induced by poor plaque control might compromise the clinical results. Longer peri-implant soft tissue around subcrestal implants might either provide a better mucosal barrier to prevent bone loss or, in contrast, favor anaerobic bacterial population retention and further contribute to bone loss under poor oral hygiene conditions. Until now, there has been little information available in the literature about whether the implant depth affects peri-implant soft tissue and bone remodeling during the development of peri-implant infections. It remains unclear whether subcrestal or equicrestal implant placement is more favorable for the long-term prognosis of dental implants (Lee et al., 2010; Romanos et al., 2015).

The experimental peri-implantitis model was developed in both dogs and monkeys and is used to evaluate the pathogenesis and treatment of peri-implantitis (Ericsson et al., 1996; Lang et al., 1993; Lindhe et al., 1992; Martinez et al., 2014). Results from recent animal studies showed that implants with different designs such as varied implant surfaces may present different peri-implant bone remodeling in the experimental peri-implantitis model (Albouy, Abrahamsson & Berglundh, 2012; Albouy et al., 2009; Fickl et al., in press). The progression of peri-implantitis was more pronounced in implants with rough surfaces compared with implants with polished surfaces when plaque formation was left untreated (Albouy, Abrahamsson & Berglundh, 2012). Implants with plasma-sprayed HA coatings were reported to be associated with marked marginal bone loss in experimental peri-implantitis (Madi et al., 2013). The results were in contrast to clinical study by Urdaneta et al. (2010) reporting implants with HA coatings can successfully function without an increased risk for peri-implant disease. Unlike implants with screwed-in tapered internal IAIs and fluoride-modified titanium dioxide grit blasted surfaces, implants with tapped-in tapered internal IAIs and plasma-sprayed calcium-phosphate surfaces were commonly inserted subcrestally in clinical practice. It was hypothesized that HA-coated implant inserted in a subcrestal position was benefit for implant success due to direct bacterial invasion of the surface might be difficult (Lee et al., 2010). However, evidence for the synergistic action of the implant surface characteristics and the insertion depth on the progress of peri-implantitis still limited.

Therefore, the aim of this study was to evaluate the impact of IAI placement depths on the peri-implant soft tissue and bone remodeling in the presence of experimentally induced peri-implant inflammation. Two different commercial implants with tapered internal IAIs and different surface characteristics were placed in dogs, and clinical and radiographic evaluations were performed. The null hypothesis was that the IAI placement depths would not affect peri-implant tissue destruction under experimentally induced peri-implant inflammation.

## Materials and Methods

### Animals

This study protocol was approved by the Medical Ethical Committee for Animal Investigations of Peking University Health Science Center in Beijing, China (Permit Number: LA2010–032). During the entire experiment, the dogs were housed individually under ambient temperature 20–25 °C, relative humidity 30–70%, and were fed once per day with soft-food diet and water ad libitum. All clinical and surgical procedures were performed under general anesthesia with intravenous sodium pentobarbital (30 mg/kg). Local anesthesia was also provided using 2% lidocaine hydrochloride with epinephrine at 1:100,000 at the surgical sites. Six male beagle dogs, 1–2 years old and weighing 10–12.5 kg, were used in this study.

### Surgery

All of the mandibular first molars and second, third, and fourth premolars were carefully extracted. After 8 weeks, implant surgery was performed. Two implants with screwed-in tapered internal IAIs and fluoride-modified titanium dioxide grit blasted surfaces (SI, OsseoSpeed, 3.5 × 8 mm; Astra Tech Dental, Mölndal, Sweden) and two implants with tapped-in tapered internal IAIs and plasma-sprayed calcium-phosphate surfaces (TI, Integra-CP, 3.5 × 8 mm; Bicon Dental Implants, Boston, Massachusetts, USA) were inserted in one side of the mandible of each dog. The anterior and posterior positions were alternated between the implant systems. A total of 24 implants were placed in the six dogs. For comparing the tissue reaction under inflamed condition with previous studies using implants with screwed-in tapered internal IAIs under non-inflamed condition (Huang et al., 2012; Weng et al., 2008), subcrestal groups were inserted 1.5 mm subcrestally. Therefore, four experimental groups were constituted: SI placed crestally (SIC); SI placed 1.5 mm subcrestally (SIS); TI placed crestally (TIC); and TI placed 1.5 mm subcrestally (TIS).

Scaling was performed to remove supragingival calculus one week before the implant surgery. For implant placement, the mucoperiosteal flaps were elevated in the edentulous region of the mandible. After the edentulous osseous ridge was carefully flattened with surgical burs, osteotomies for implants were drilled following the manufacturers’ recommendations. Implants representing each group were inserted. For standardizing the depth of subcrestal implant, vertical distance between crestal bone and IAI was measured using a periodontal probe (UNC-15; Hu-Friedy, Chicago, Illinois, USA) during implant placement. The depth of implant placement was further confirmed by standardized periapical radiographs. The distal implant was placed 10 mm from the second molar, and a distance of ≈10 mm was maintained between the dental implant centers to avoid interactions among the bone defects. Cover screws and/or plug inserters were placed, and flaps were sutured with 4-0 nylon sutures to submerge all implants. Antibiotics (penicillin G procaine, 40,000IU/kg, intramuscularly) and analgesics were administered once per day for one week. The sutures were removed 10 days after implant placement.

After 12 weeks of healing, all 24 implants were exposed by minimal invasion and were connected with commercially available healing abutments. For the SI implants, 4.5 mm × 4 mm and 4.5 mm × 6 mm healing abutments were used in the crestal and subcrestal groups, respectively. For the TI implants, 4.0 mm × 4.5 mm and 4.0 mm × 6.5 mm healing abutments were used in the crestal and subcrestal groups, respectively. Attention was taken to avoid occlusal contact. The exposed abutments and peri-implant soft tissues were irrigated with 0.12% chlorhexidine digluconate every second day for the first 10 days after surgery. After that, an oral hygiene procedure using a soft toothbrush with 0.2% chlorhexidine gel was performed every second day.

### Experimental peri-implantitis

Four weeks after abutment connection, plaque control was terminated and cotton ligatures were placed in a submarginal position around the abutments to promote plaque accumulation and induce plaque-associated peri-implant inflammation as methods described by Tillmanns et al. (1998). The ligatures were checked once per week without forcing them into an apical position. Plaque accumulation was allowed to continue for 12 weeks.

### Clinical evaluation

**Figure 1:**
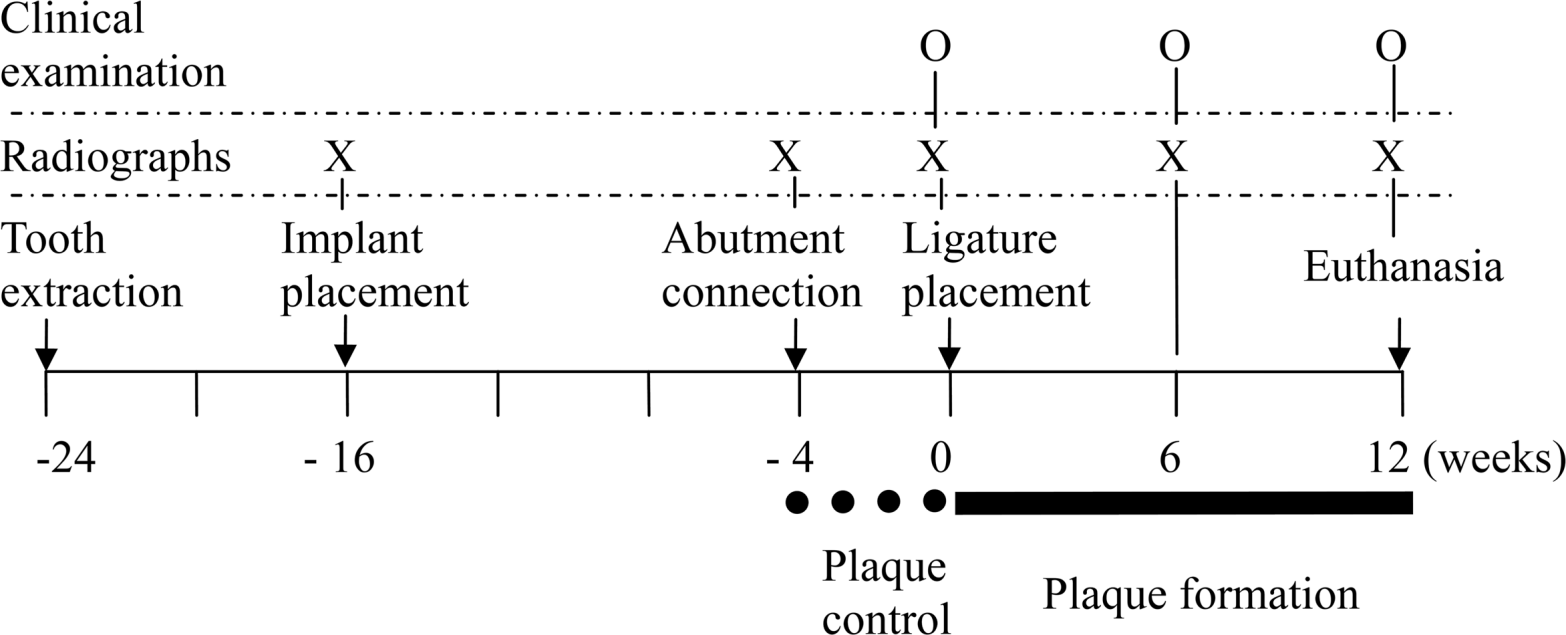
Outline of the study.

Baseline clinical measurements were taken at the mesial and distal sites of each implant before placing the ligatures. Using a periodontal probe (UNC; Hu-Friedy, Chicago, Illinois, USA), the peri-implant probing depths (PD, distance from the gingival margin to the bottom of the sulcus/pocket) and clinical attachment levels (CAL, distance from fixed point in the abutment shoulder to the bottom of the sulcus/pocket) were measured. In addition, the modified plaque index (PI) (Mombelli et al., 1987) and bleeding index (BI) (Mazza, Newman & Sims, 1981) were measured. Clinical measurements were repeated at 6 and 12 weeks after ligature placement (Fig. 1). CALs were adjusted by the different abutment lengths among the groups. All clinical measurements were performed by one calibrated examiner.

### Radiographic analysis

**Figure 2:**
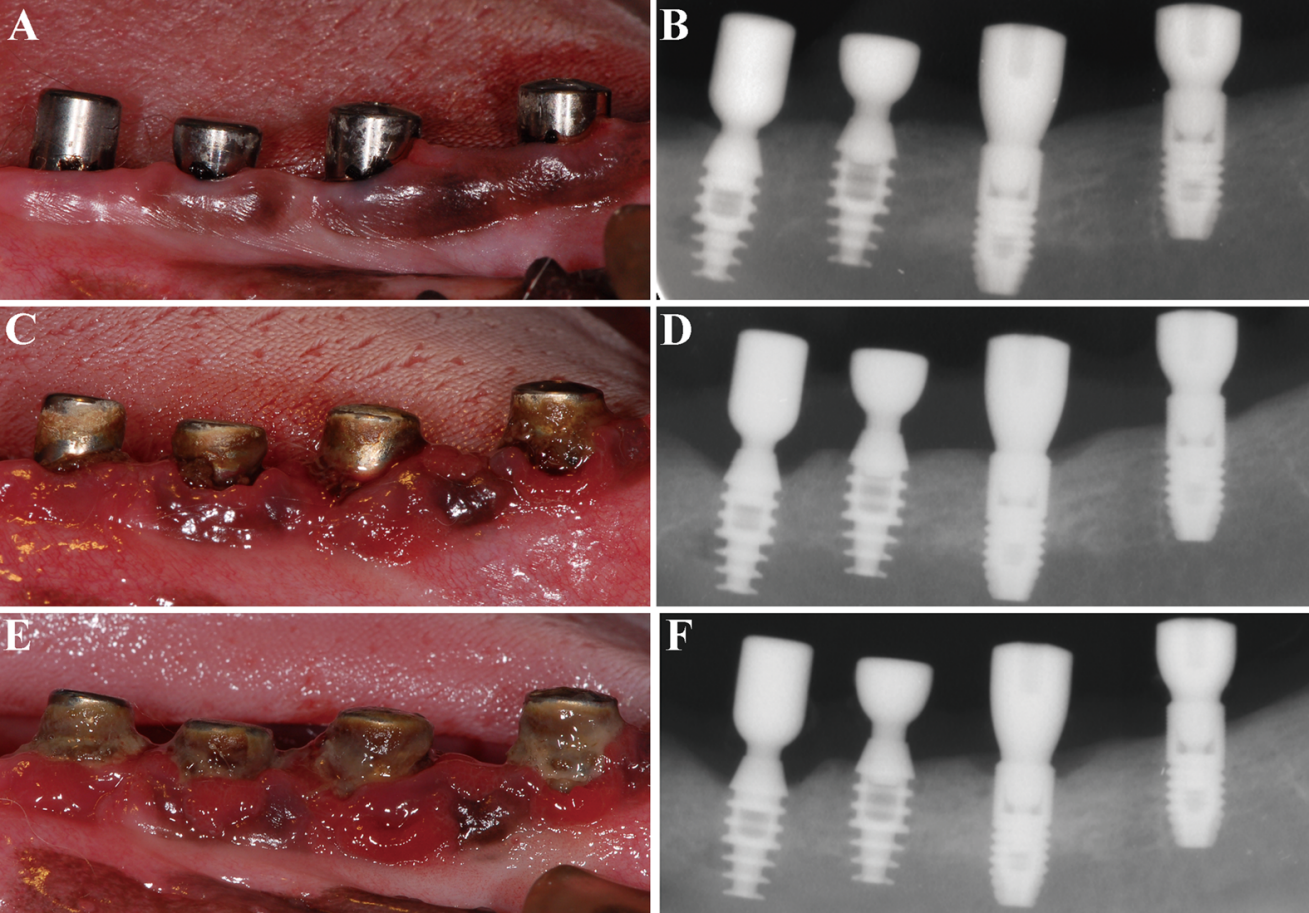
Clinical and radiographic images of the four groups. (A) and (B) at ligature placement; (C) and (D) 6 weeks after ligature placement; (E) and (F) 12 weeks after ligature placement. From left to right: TIS, TIC, SIS and SIC, respectively.

Before radiographic evaluation, radiographic templates were fabricated, and an optimum standardized parallel radiographic technique was created to minimize angulation and distortion errors (Huang et al., 2012). Standardized periapical radiographs were taken with a digital image system (Digora; Soredex, Helsinki, Finland) at baseline (ligature placement) and at 6 and 12 weeks after ligature placement (Fig. 2). The exposure parameters were 60 kV, 7 mA, and 0.16 s at a focus-film distance of 37 cm.

**Figure 3:**
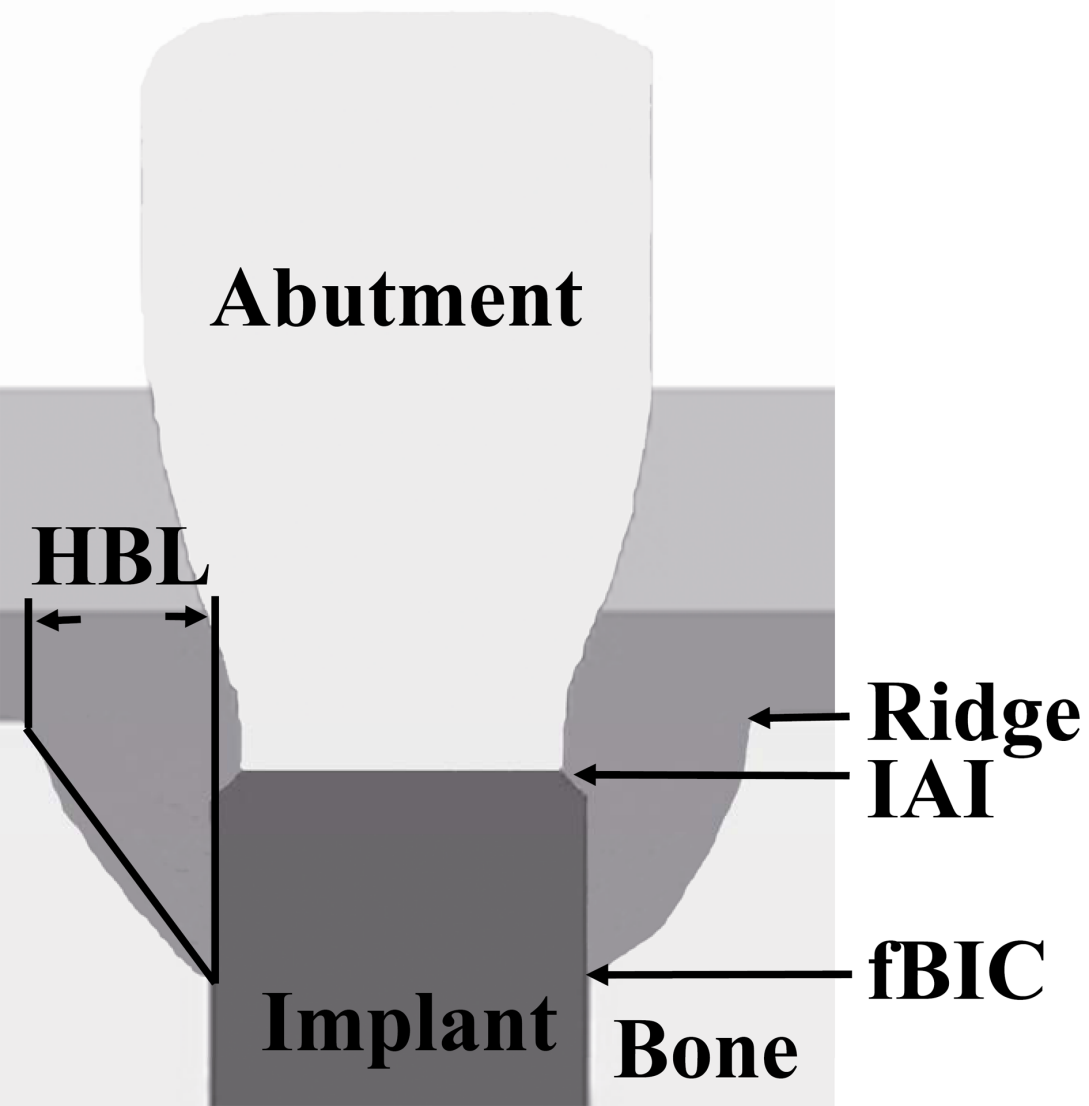
Schematic representation of the landmarks for the measured radiographic parameters. (1) Ridge; (2) IAI; (3) fBIC; (4) HBL.

The following parameters were measured at the mesial and distal sites of each implant (Fig. 3): (1) vertical bone level, measurement from the IAI to the first bone-implant contact (IAI-fBIC); in situations where the marginal hard tissue was observed above the IAI, it was still recorded as 0 to avoid introducing any bias into the results; (2) ridge loss, vertical measurement from the ridge to the IAI 10 days after implant placement (original ridge) minus the follow-up measurement from the ridge to the IAI; (3) ridge-fBIC, vertical measurement from the ridge to the fBIC, which represents the depth of the infrabony defect; (4) horizontal bone loss (HBL), horizontal measurement from the peri-implant ridge to within 3 mm of the implant body. The measurements were adjusted for distortion using the total length of the implant. The mesial and distal values were averaged to obtain a mean value for each implant. A software program (NIH Image J v.1.44n; National Institutes of Health, Bethesda, Maryland, USA) was used to analyze each calibrated image. Radiographic image alignment and analysis were performed by one calibrated examiner.

### Statistical analysis

Intra-examiner reliability was determined by calculating the standard error of measurement (SE) and Spearman correlation coefficient (CC) for clinical (SE = 0.31 mm; CC = 0.889) and radiographic (SE = 0.11 mm; CC = 0.983) measurements.

SPSS (SPSS 18.0, Chicago, Illinois, USA) and R software (version 3.0.1; R foundation for Statistical Computing, Vienna, Austria) were used for data analyses, including values for the mean, median, standard deviation (SD), and the range from the lower quartile (25th percentile) to the upper quartile (75th percentile). The R-library “nparLD 2.1” (Noguchi et al., 2012) was used to perform the Brunner–Langer nonparametric analysis of longitudinal data in factorial experiments (Brunner, Domhof & Langer, 2002). The effects of IAI placement depth, IAI type, and their interaction on clinical and radiographic parameters were assessed. The null hypothesis was rejected at *P* ≤ 0.05.

## Results

During the experiment, healing was uneventful around all implants. Clinically healthy peri-implant mucosa was observed around each implant before ligature placement. Plaque formation after ligature placement resulted in marked signs of inflammation around the implants. At 12 weeks after ligature placement, the mean PI was 2.8 and the mean BI was 3.8. No statistically significant differences were found among the 4 groups.

### Clinical findings

**Figure 4:**
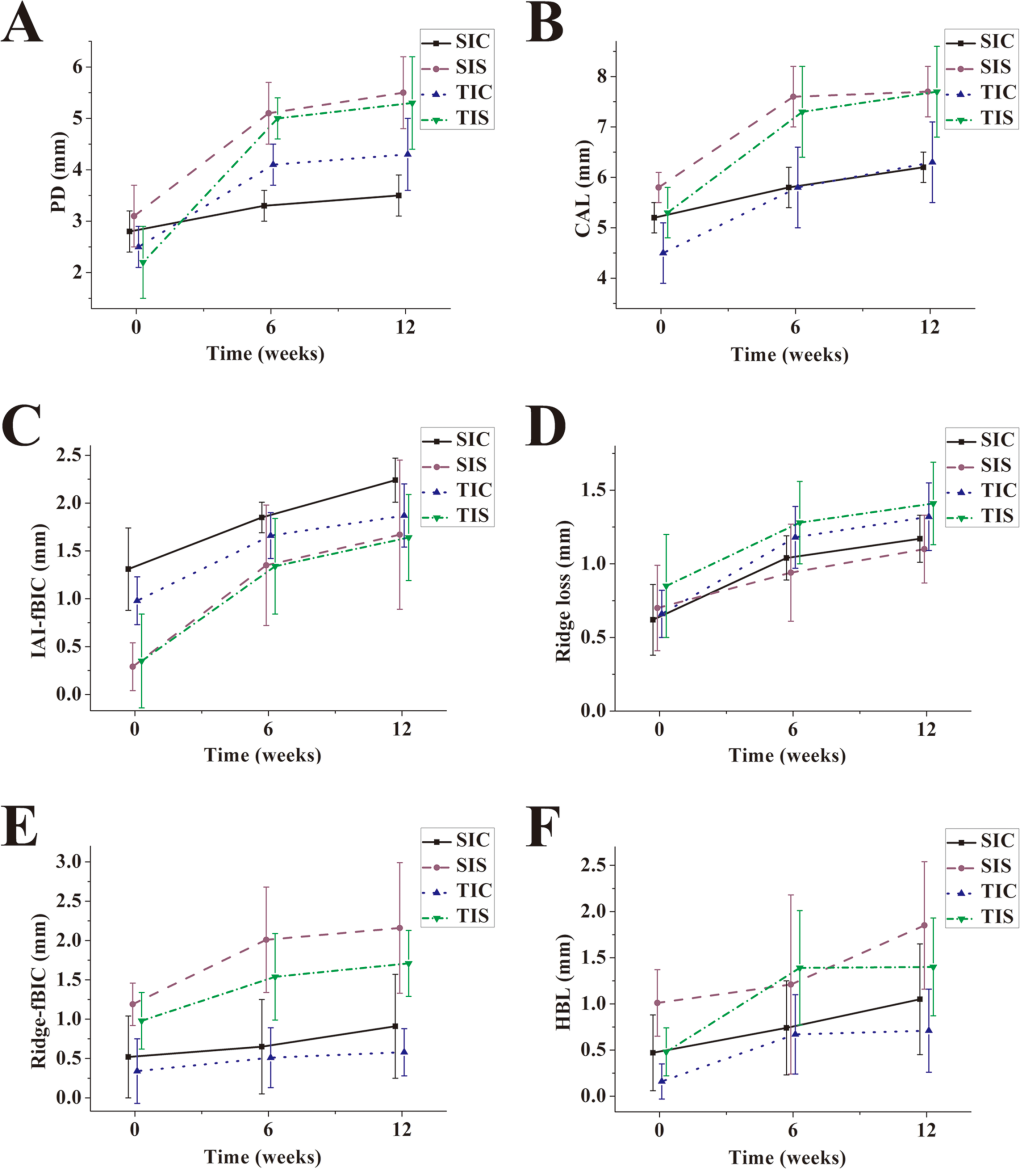
Plotting of the means of clinical and radiographic parameters for all groups after ligature-induced peri-implant infection. (A) PD; (B) CAL; (C) IAI-fBIC; (D) Ridge loss; (E) Ridge-fBIC; (F) HBL.

The means of PD and CAL of all groups were statistically increased during the dynamic period when comparing the baseline data with 6-week and 12-week data, respectively (*P* < 0.05) (Figs. 4A and 4B). After ligature placement, the PD alterations depended on the IAI placement depth (*P* < 0.001) and IAI type (*P* = 0.010) but not on the interaction between the IAI placement depth and the IAI type (*P* = 0.953) (Tables 1 and 2). PD alterations in the subcrestal groups were significant larger than that in the equicrestal groups. PD alterations in the TI groups were significantly greater than those in the SI groups. The CAL alterations depended on the IAI placement depth (*P* < 0.001) but not on the IAI type (*P* = 0.101) or the interaction between the IAI placement depth and the IAI type (*P* = 0.523).

**Table 1:** Descriptive statistics for the clinical and radiographic parameter alterations during the 12-week period following ligature placement.^a^

Parameter		Crestal		Subcrestal	
		SI (SIC)	TI (TIC)	SI (SIS)	TI (TIS)
ΔPD	Mean ± SD	0.7 ± 0.4	1.9 ± 1.0	2.4 ± 1.0	3.2 ± 1.1
	Median (Q1, Q3)	0.8 (0.4, 1.0)	1.8 (1.0, 2.6)	2.7 (1.6, 3.1)	3.1 (2.2, 4.1)
ΔCAL	Mean ± SD	1.0 ± 0.4	1.7 ± 1.0	2.0 ± 0.6	2.5 ± 1.1
	Median (Q1, Q3)	1.0 (0.7, 1.2)	1.9 (0.8, 2.6)	1.9 (1.6, 2.6)	2.5 (1.6, 3.3)
ΔIAI-fBIC	Mean ± SD	0.94 ± 0.44	0.90 ± 0.41	1.38 ± 0.70	1.29 ± 0.51
	Median (Q1, Q3)	0.86 (0.57, 1.30)	1.08 (0.48, 1.20)	1.28 (0.77, 1.98)	1.34 (1.00, 1.69)
ΔRidge loss	Mean ± SD	0.55 ± 0.18	0.66 ± 0.27	0.41 ± 0.19	0.56 ± 0.14
	Median (Q1, Q3)	0.54 (0.42, 0.70)	0.55 (0.46, 0.97)	0.37 (0.26, 0.55)	0.54 (0.46, 0.67)
ΔRidge-fBIC	Mean ± SD	0.39 ± 0.39	0.24 ± 0.36	0.97 ± 0.85	0.73 ± 0.47
	Median (Q1, Q3)	0.32 (0.10, 0.66)	0.14 (0.07, 0.66)	0.89 (0.14, 1.74)	0.72 (0.42, 1.19)
ΔHBL	Mean ± SD	0.59 ± 0.55	0.55 ± 0.45	0.84 ± 0.91	0.92 ± 0.57
	Median (Q1, Q3)	0.51 (0.11, 1.01)	0.51 (0.23, 0.80)	0.62 (0.10, 1.69)	0.67 (0.48, 1.59)

**Notes.**

a *n* = 6. Units: mm.

ΔPD peri-implant probing depth alteration ΔCAL clinical attachment level alteration ΔIAI-fBIC vertical bone level alteration ΔRidge loss ridge loss alteration ΔRidge-fBIC depth of infrabony defect alteration ΔHBL horizontal bone loss alteration SD standard deviation Q1 first quartile Q3 third quartile SI screwed-in IAI TI tapped-in IAI

**Table 2:** Effects of IAI placement depth, IAI type, and their interaction on clinical and radiographic parameter alterations during the 12-week period following ligature placement.

	IAI placement depth (Crestal vs. Subcrestal)		IAI type (SI vs. TI)		IAI placement depth × IAI type	
	Statistic^a^	*P* value	Statistic^a^	*P* value	Statistic^a^	*P* value
ΔPD	19.794	<0.001	6.648	0.010	0.961	0.327
ΔCAL	25.867	<0.001	2.685	0.101	0.407	0.523
ΔIAI-fBIC	3.684	0.05	0.044	0.833	0.062	0.803
ΔRidge loss	3.503	0.061	2.955	0.086	0.796	0.372
ΔRidge-fBIC	4.651	0.031	0.476	0.490	0.033	0.856
ΔHBL	0.627	0.428	0.241	0.624	0.723	0.395

**Notes.**

a ANOVA-Type Statistics (ATS).

ΔPD peri-implant probing depth alteration ΔCAL clinical attachment level alteration ΔIAI-fBIC vertical bone level alteration ΔRidge loss ridge loss alteration ΔRidge-fBIC depth of infrabony defect alteration ΔHBL horizontal bone loss alteration. SD standard deviation Q1 first quartile Q3 third quartile SI screwed-in IAI TI tapped-in IAI

*n* = 6. Units: mm.

### Radiographic findings

Vertical bone level (IAI-fBIC) alterations following the ligature-induced plaque accumulation period depended on the IAI placement depth (*P* = 0.05) but not the IAI type (*P* = 0.833) or the interaction between the IAI placement depth and the IAI type (*P* = 0.803) (Tables 1, 2 and Fig. 4C). IAI-fBIC alterations in the subcrestal groups were significant larger than those in the crestal groups.

Regarding the configuration of the bone defects, the depths of infrabony defect (Ridge-fBIC) alterations in the subcrestal groups were significant larger than those in the crestal groups (*P* = 0.031) (Tables 1 and 2). Alterations of supra-alveolar bone loss (ridge loss) and horizontal bone loss (HBL) were comparable in all groups after ligature placement (*P* > 0.05) (Figs. 4D–4F).

## Discussion

The present study was designed to evaluate the influence of implant placement depth on peri-implant soft and hard tissues in the early stages of peri-implantitis in a canine model. To the best of our knowledge, the effect of implant placement depth on peri-implant bone remodeling under inflamed conditions has not been previously studied. Based on the results of this study, the null hypothesis was rejected. In the presence of experimentally induced peri-implant inflammation, the alterations of PD, CAL, IAI-fBIC and Ridge-fBIC were significant larger in the subcrestal groups compared with the crestal groups (*P* < 0.001, *P* = 0.05 and *P* = 0.031, respectively).

A ligature-induced peri-implant infection animal model was used in the present study, which was widely applied in studying the pathogenesis and treatment of peri-implantitis (Ericsson et al., 1996; Fickl et al., in press; Lang et al., 1993; Lindhe et al., 1992; Martinez et al., 2014). After ligature placement, subgingival bacterial accumulation led to inflammation, pocket formation and peri-implant bone loss. In this study, the ligatures were placed slightly below the mucosal margin and were not exchanged or added during the experiment period to reduce the influence of the operator on the placement depth of the ligature. Furthermore, an early ligature implantation protocol at 4 weeks after abutment connection was used in present study. It was comparable with conventional model in which 12 weeks healing time was allowed and it was useful for evaluating the pathogenesis of peri-implantitis (Martinez et al., 2014; Park et al., in press). Another animal model was used to mimic the spontaneous progression of peri-implantitis, in which the ligature was removed after the establishment of peri-implantitis (Zitzmann et al., 2004). This seems to be a valuable model for studying the progression of peri-implantitis to more advanced stages. In the present study, the ligature was kept in contact with the peri-implant tissue during the whole experimental period, and no spontaneous progression was allowed; this allowed for the evaluation of the early reaction of the peri-implant tissues in the presence of plaque accumulation.

In present study, peri-implant probing provoked significant bleeding under ligature-induced peri-implantitis, which was unrelated to the probing depth. This finding was in agreement with Madi’s study (Madi et al., 2013). It was suggested that the absence of bleeding is a reliable predictor of peri-implant health (Salvi & Lang, 2004), but the extent of bleeding was not associated with the amount of inflammation in peri-implant soft tissues (Martins et al., 2004; Schou et al., 2002).

In this experiment, the alterations of PD and CAL in the subcrestal groups were significant larger than that in the equicrestal groups after ligature placement. Previous studies reported the epithelial dimension and biologic width were significantly greater for implants placed subcrestally compared to implants placed crestally and the connective tissue dimension was not different (Cochran et al., 2013; Huang et al., in press). Because the probes were able to identify the connective tissue adhesion level in the healthy group (Lang et al., 1994), the baseline values of PD and CAL were larger in subcrestal groups compared with crestal groups. Since periodontal probe may penetrate beyond the apical termination of the junctional epithelium and reach a level close to the bone crest after ligature-induced inflammation (Lang et al., 1994), it can not definitely eliminate the influence of the difference of biological width between subcrestal group and crestal group on the values of PD and CAL after ligature placement. However, due to connective tissue dimension was not different between subcrestal groups and crestal groups (Cochran et al., 2013; Huang et al., in press) and probe penetration was increased with the degree of inflammation (Lang et al., 1994), the alterations of PD and CAL found in present study were chiefly attributed to the soft tissue inflammation and bone loss. More severe bone loss in the subcrestal groups compared with the crestal groups after ligature placement was observed. The results of present study implied that the absolute values of PD and CAL loss were influenced by the dental implant placement depth. The PD and CAL loss values were significantly increased for all groups after ligature placement, especially for the subcrestal groups. Therefore, the establishment of baseline PD and CAL values was important to allow for comparison with future examinations.

In the present experiment, the subcrestal groups had lower IAI-fBIC compared with the crestal groups at baseline (Fig. 3C), which was consistent with previous report (Huang et al., 2012). However, at the end of the ligature-induced peri-implant inflammation period, IAI-fBIC around the crestal and subcrestal implants was not significantly different. The results showed that the amount of bone loss that occurred during the plaque accumulation period after ligature placement was significantly larger at subcrestal implants than at crestal implants (*P* = 0.05), implying that implant placement depth might influence the progression of peri-implant inflammation in the early stages of peri-implantitis.

Previous studies have showed that subcrestal placement of tapered internal IAI had a positive impact on crestal bone preservation around the cervix of the implant (Barros et al., 2010; Donovan et al., 2010; Huang et al., 2012). Despite the good results obtained under non-inflamed conditions, this study showed that subcrestal implants exhibited more severe soft tissue destruction and bone loss during the ligature-induced peri-implant infection. Since tapped-in and screwed-in internal conical abutment connection could prevent or minimize the bacterial leakage along the IAI (Dibart et al., 2005; Aloise et al., 2010), the role of microleage in IAI of subcrestal groups seems not the main reason for this phenomenon. It was reasonable to assume that the peri-implant soft tissue around implant inserted in subcrestal positions is more sensitive to the irritation from bacterial plaque compared with implant inserted in crestal positions, although the quality and quantity of bacterial plaque have not been fully understood. It was reported that the epithelial dimension and biologic width were significantly greater for subcrestal implants compared with crestal implants (Cochran et al., 2013; Huang et al., in press). Epithelial sealing is one of the most fragile points of peri-implant tissue integration. After ligature-induced plaque accumulates on the implant abutment, the following loss of epithelial sealing allows the plaque front to continue to apically migrate (Lang et al., 1994; Lindhe et al., 1992). Therefore, the more significant peri-implant pockets in the subcrestal implants favored the accumulation of periodontal pathogenic bacterial species. Renvert et al. (2007) collected clinical and microbiological data from 213 subjects with 976 functional implants and found that implant probing pocket depth at the implant site with the deepest probing depth was correlated with levels of several periodontal pathogenic bacterial species. Another study also showed a positive correlation between the presence of highly pathogenic bacteria and the pocket depth (Al-Radha et al., 2012). Moreover, the quality and quantity of the bacterial attack are closely associated with the severity of peri-implant destruction (Lang et al., 1994). Therefore, further microbiologic analysis should be conducted to verify the relationship between microbiota and peri-implant bone loss in crestal and subcrestal implants.

After 12 weeks of ligature-induced peri-implantitis, the IAI-fBIC of the implants ranged from 1.64 mm to 2.24 mm, which was comparable between the subcrestal and crestal groups (Fig. 3C). Bone loss in the crestal groups was in accordance with the results of the animal study by Tillmanns et al. (1998), who reported vertical bone loss between 2.53 and 2.90 mm around implants with three different surfaces after 3 months of ligature-induced peri-implantitis. However, less vertical bone loss was observed in the crestal groups compared with that observed by Martins and co-workers (Martins et al., 2004), who reported approximately 6 mm of vertical bone loss 60 days after ligature placement, and Albouy et al. (2008), who reported bone level alterations ranging from 3.53 to 4.69 mm during a 12 week period of ligature-induced “active breakdown.” A discrepancy in the methodologies might explain the differences between these results. In the study of Martins et al. (2004), further ligatures were placed at 20-day intervals for a period of 60 days, whereas in the study of Albouy et al. (2008), the ligatures were replaced at 3-week intervals. The more apical location of the ligature might also contribute to the greater bone loss observed compared with the present study.

In this study, the alterations of ridge-fBIC after ligature placement were more obvious in subcrestal implants compared with crestal implants. This result showed that the difference in bone defect configurations between crestal and subcrestal implants tended to broaden under ligature-induced peri-implant inflammation. The relationship between this difference and the long-term success of the dental implant was not clear. The limited available clinical data were inconsistent regarding the influence of subcrestal versus crestal placement of implants with tapered internal IAIs on the long-term prognosis of the dental implant. In a retrospective study, Lee et al. (2010) showed that the installation depth was an important prognostic factor in HA-coated implants with tapped-in tapered internal IAIs. The failure rate for the HA-coated implants installed at the margin level was significant larger than that for implants placed 2 mm subcrestally. Conversely, in another recent retrospective study by Romanos et al. (2015), bone losses were comparable between subcrestal and crestal implants with screwed-in tapered internal IAIs and sandblasted, acid-etched surfaces. From a clinical point of view, the depth of the infrabony defects might affect the difficulty of peri-implant debridement through a non-surgical treatment approach during peri-implantitis treatment, and the surgical regenerative treatment approach might also be influenced by the peri-implant defect configuration (Schwarz et al., 2010). Further clinical studies are suggested to evaluate the relationship between the IAI placement depth and the long-term success of dental implants.

Several factors should also be taken into consideration as causative factors of peri-implant bone remodeling under inflamed conditions, including implant geometry, implant surface characteristics, bone qualities and quantities, all of which could have an effect (Albouy et al., 2008; Serino & Turri, 2011). Recently, researchers have focused on studying exactly how implant surface characteristics contribute to the development of peri-implant disease (Albouy, Abrahamsson & Berglundh, 2012; Fickl et al., in press; Madi et al., 2013). Studies showed that implant surface characteristics might influence the spontaneous progression and the treatment outcome of peri-implantitis (Albouy, Abrahamsson & Berglundh, 2012; Albouy et al., 2011; Madi et al., 2013). In the present study, two types of implants with tapered internal IAIs were used. The alterations of clinical and radiographic parameters after ligature placement were comparable, which implied that both commercial implant types had similar soft and hard tissue responses under conditions of inflammation.

Notably, this study was conducted under unloaded conditions. Some studies have shown that overloading increases bone resorption in the presence of plaque-induced inflammation (Chambrone, Chambrone & Lima, 2010; Kozlovsky et al., 2007). Another limitation of the present study was that the bone levels in the buccal and lingual orientations might not be exactly the same as those in the mesial and distal orientations. However, this cannot be determined by two-dimensional X-rays. Further histological study is required to clarify this issue. Furthermore, implants inserted in supercrestal level were not evaluated in this study. Recent study showed that supracrestal placement of implant was associated with less bone loss compared with crestal placement of implant (Van Eekeren, Tahmaseb & Wismeijer, in press). The peri-implant tissue reactions of supercrestal implant with ligature-induced peri-implantitis could not be drawn out in this experiment. Despite its preliminary nature, this study indicates that, under inflamed conditions, the placement depth of a dental implant affects the peri-implant probing depth, clinical attachment level and bone loss.

## Conclusion

Within the limits of this study, it was concluded that tissue destruction in subcrestal implants may be more serious than that in crestal implants in the presence of inflamed peri-implant mucosa.

## Supplemental Information

10.7717/peerj.1139/supp-1 Supplemental Information 1 Raw data
